# Supplementary figures and images for: Single cell RNA-sequencing reveals no evidence for meiotic sex chromosome inactivation in the threespine stickleback fish
Source: PLoS Genet. 2025 Sep 29;21(9):e1011875. doi: 10.1371/journal.pgen.1011875 (PMC12503298; doi:10.1371/journal.pgen.1011875)

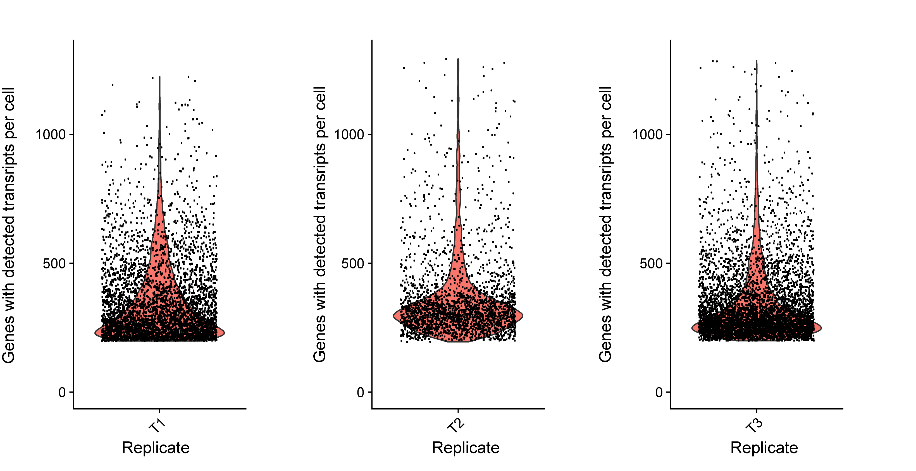

Supplement: S1 Fig — Number of transcripts detected for each gene was totaled for each cell barcode within each of the three single cell RNA sequencing experiments. A similar distribution of detected genes were identified in each of three experiments. (TIF) [file pgen.1011875.s001.tif]

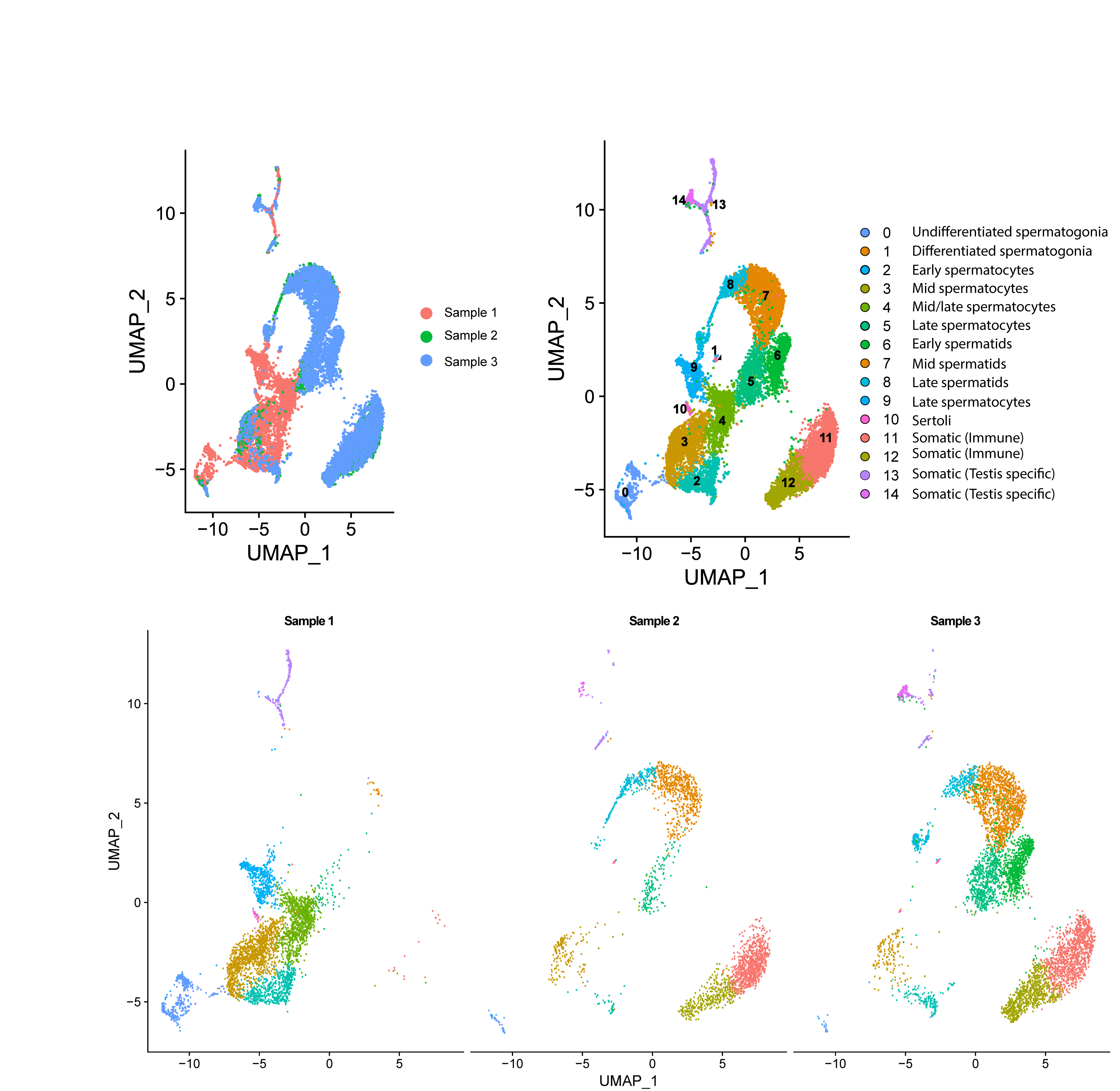

Supplement: S2 Fig — Cell barcodes from all three sequencing runs were merged, and UMAP is depicted for all merged cells (top), and for each replicate separately (bottom). Proportion of each cell type within each sample was dependent on the onset of synchronous spermatogenesis in each individual. Only cell clusters identified in two of three replicates were used for downstream analysis. (TIF) [file pgen.1011875.s002.tif]

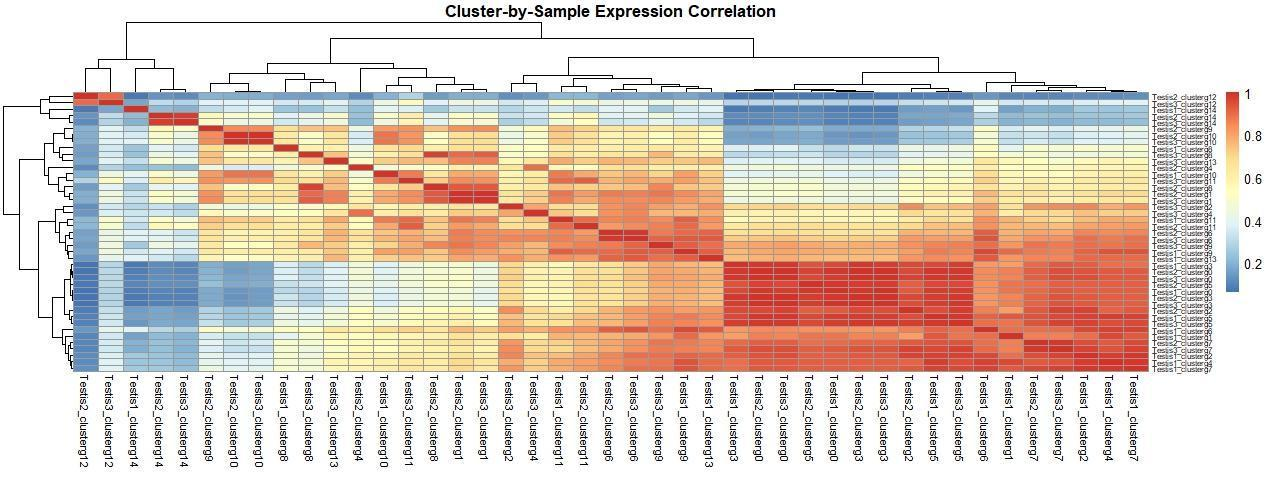

Supplement: S3 Fig — Normalized gene expression of the top 500 expressed genes in each cluster was averaged and compared for each cluster and sample. Hierarchical clustering reveals that most clusters cluster by cell types across replicates rather than by sample. (TIF) [file pgen.1011875.s003.tif]

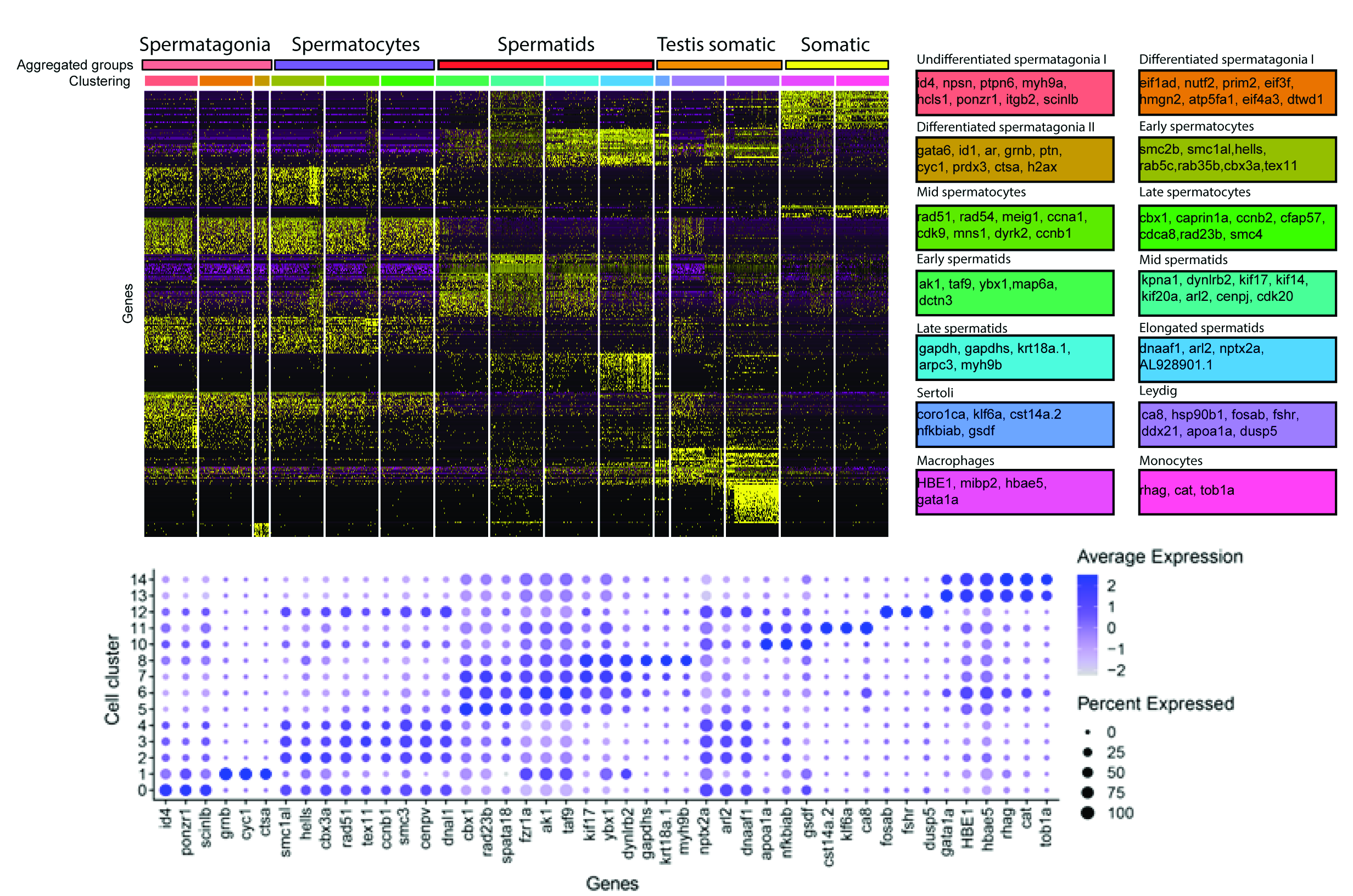

Supplement: S4 Fig — Differentially expressed genes in each cell cluster correspond with diverse testis cell types. Heatmap was generated for the top 250 variable expressed genes across all clusters. Represented marker genes for each cluster are listed in boxes. Clusters were assigned to five major cell groups for downstream analysis.based on similar expression and biological functions. Bottom: Cluster dotplots for representative marker genes. Marker genes were identified based on having significantly different expression (P < 0.01, Mann-Whitney U test), and had a confirmed function in the cell type in other species. (TIF) [file pgen.1011875.s004.tif]

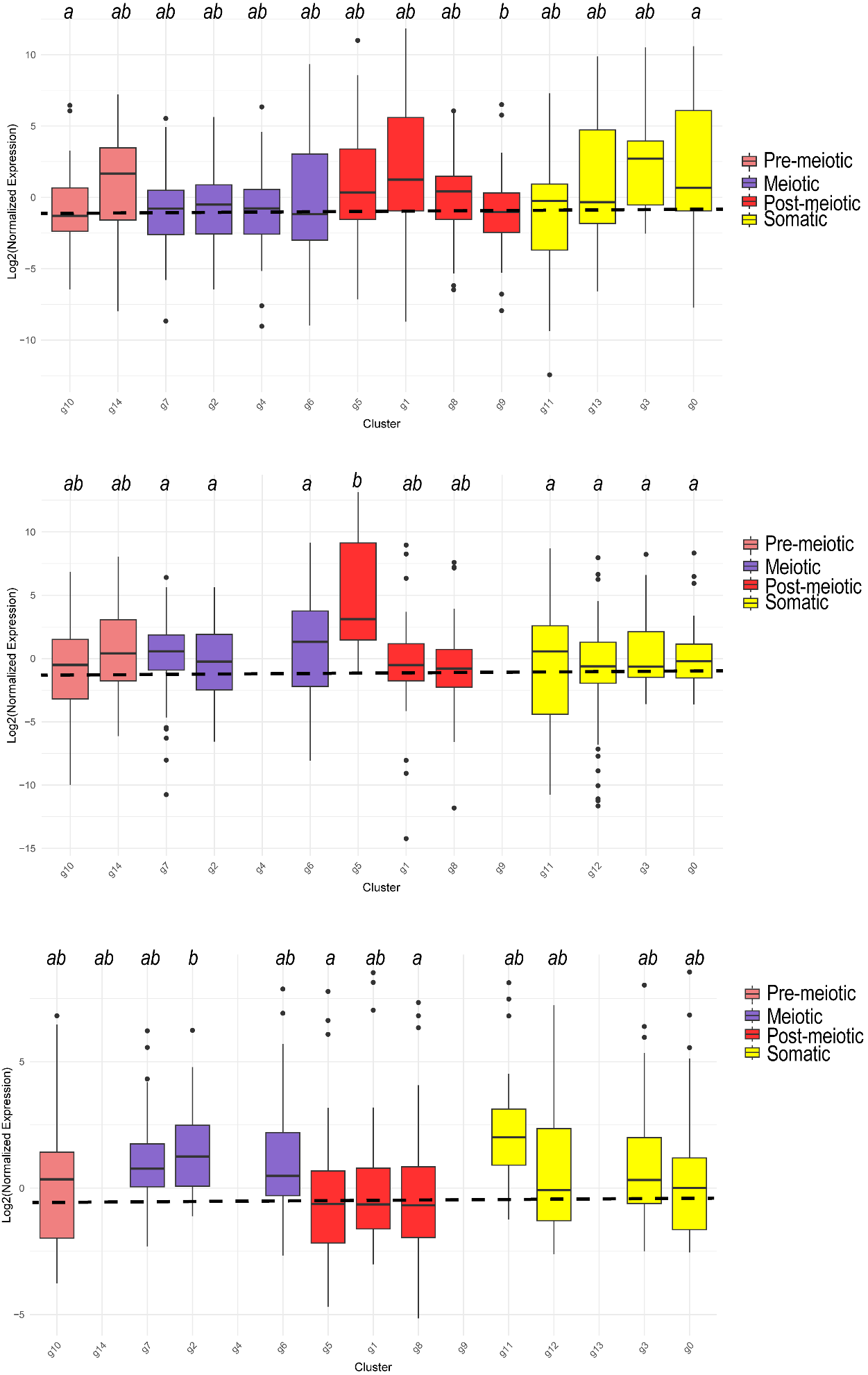

Supplement: S6 Fig — No evidence for MSCI across all three samples when examining all identified clusters. X gene expression was normalized by autosomal gene expression for each cluster across all three samples. Letters denote statistical significance based on a Kruskal Wallis test and Dunn’s test. Dotted line represents median X:A expression level across main cell groups in Fig 2. (TIF) [file pgen.1011875.s006.tif]

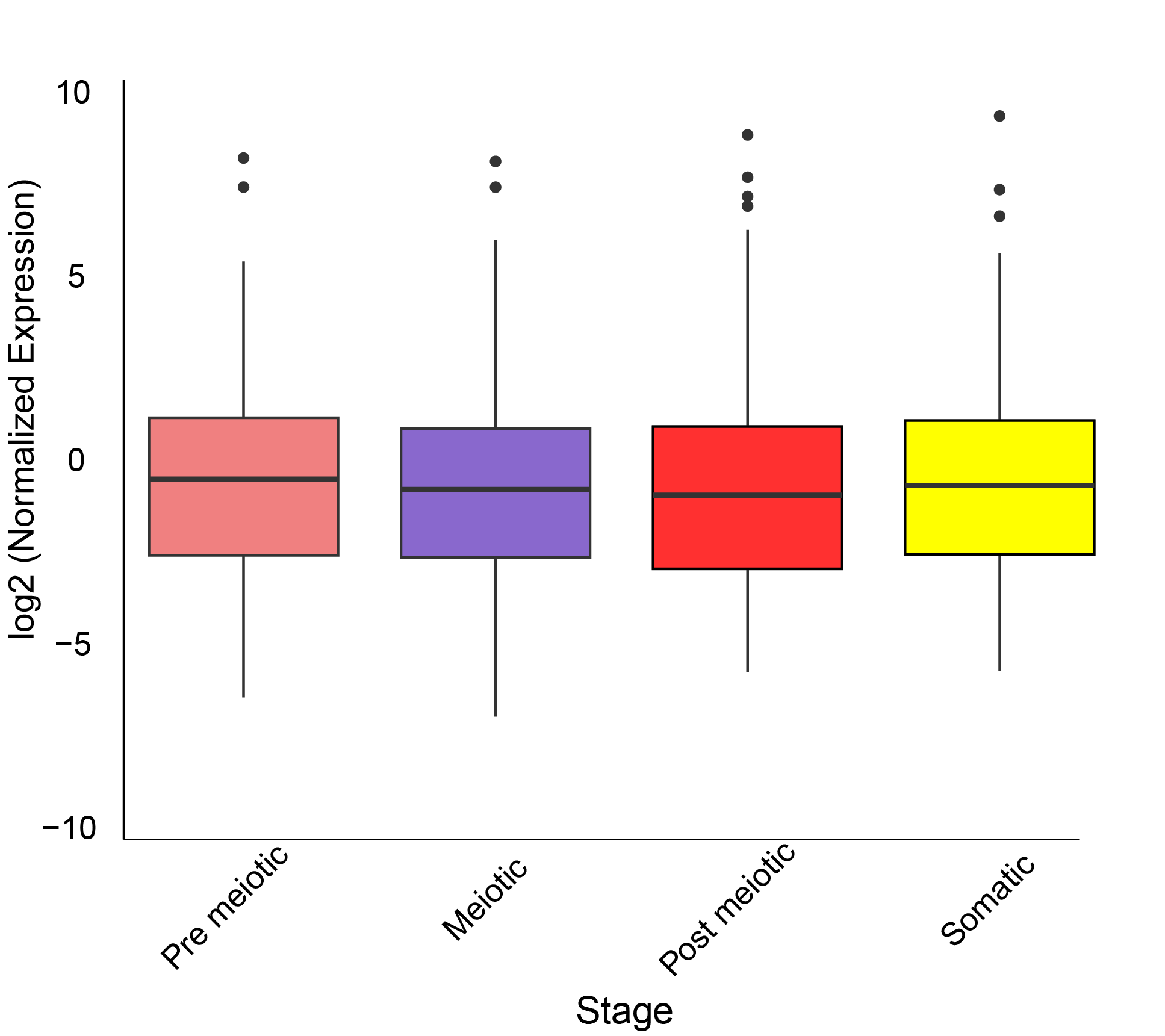

Supplement: S7 Fig — Bulk RNA-seq data from three tissues brain, liver, and testis were compared to identify genes that were expressed testis specifically. When we filtered out 20 testis specific X-linked genes, we still identify similar expression across all stages of spermatogenesis P > 0.05 for all comparisons, Kruskal-Wallis and Dunn’s test. (TIF) [file pgen.1011875.s007.tif]

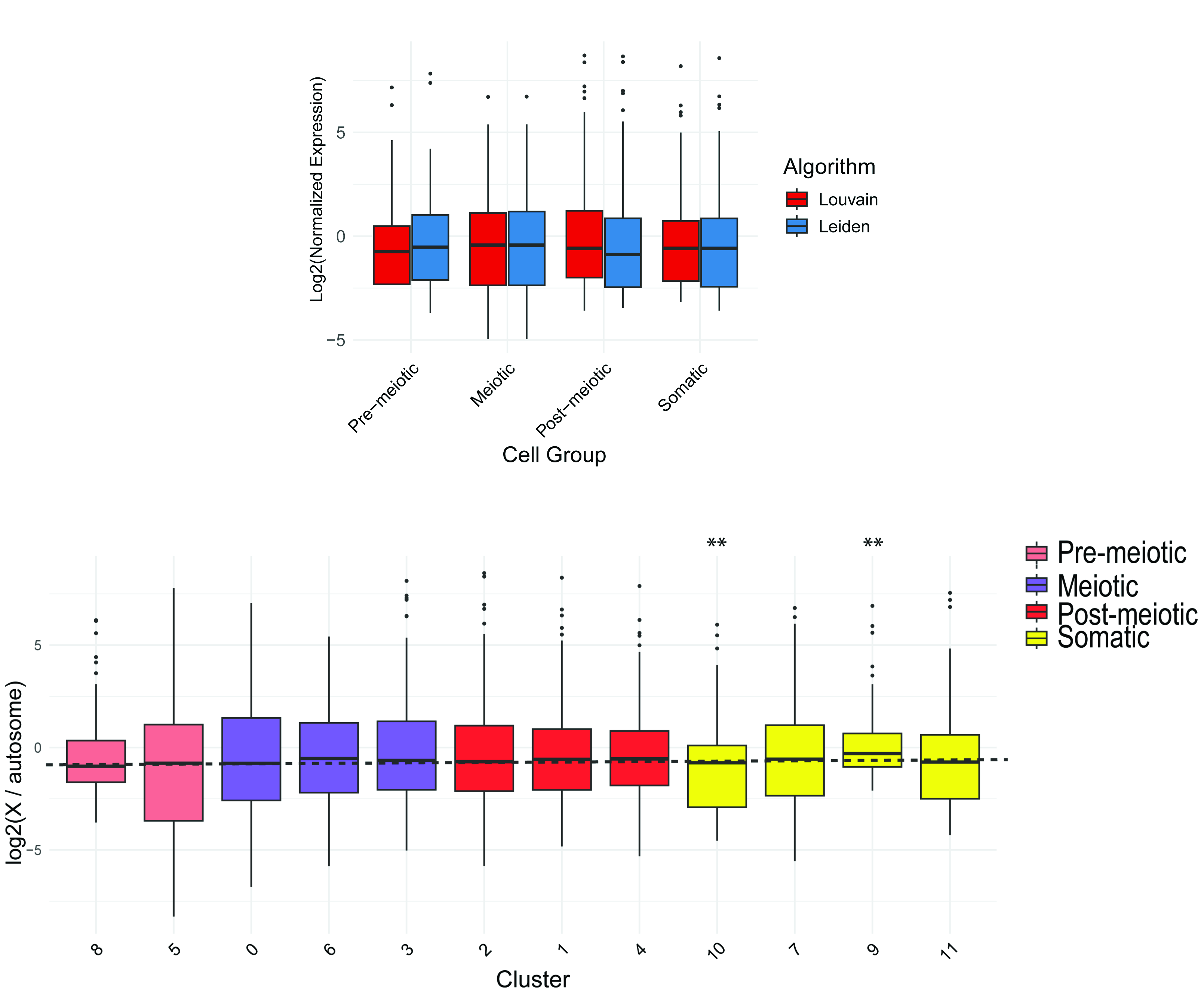

Supplement: S8 Fig — Top: Normalized X expression was compared between two distinct clustering algorithms in Seurat. X expression was similar across major cell groups for both algorithms (P = 0.441, Kruskal Wallis test. Bottom: Cell clustering was reanalyzed with a separate software package (scanpy) and re-annotated using cell markers. Scanpy clustering was performed using all three samples. Normalized X expression is similar across all stages of spermatogenesis. Only two somatic clusters had variable expression (P < 0.05, Kruskal wallis and Dunn’s test). (TIF) [file pgen.1011875.s008.tif]

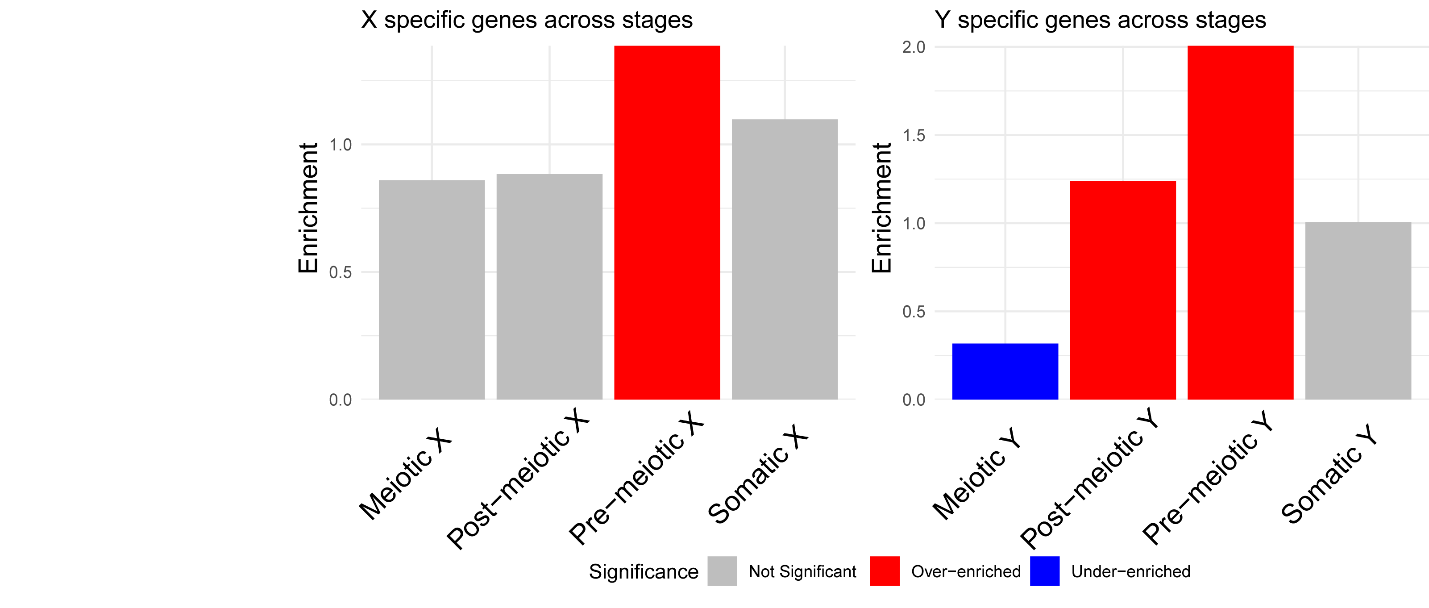

Supplement: S9 Fig — Gametolog specific expression was identified for the 4 main cell types of spermatogenesis by aggregated counts from all clusters. Genes with transcripts aligned to only one sex chromosome per cluster were identified. Mono-allelic expression was confirmed using bulk RNA-seq from three replicates of meiotically active testis. Enrichment of mono-allelic expression across cell stages was calculated using a hypergeometirc test comparing the number of mono-allelic expressed genes observed to expected based on the number of genes expressed in that cell type. X- only expression was overenriched in pre-meiotic cells (P < 0.01). Y-only expression was over-enriched in pre-meiotic and post-meiotic cell types (P < 0.01), and under-enriched in meiotic cell types (P < 0.01). (TIF) [file pgen.1011875.s009.tif]

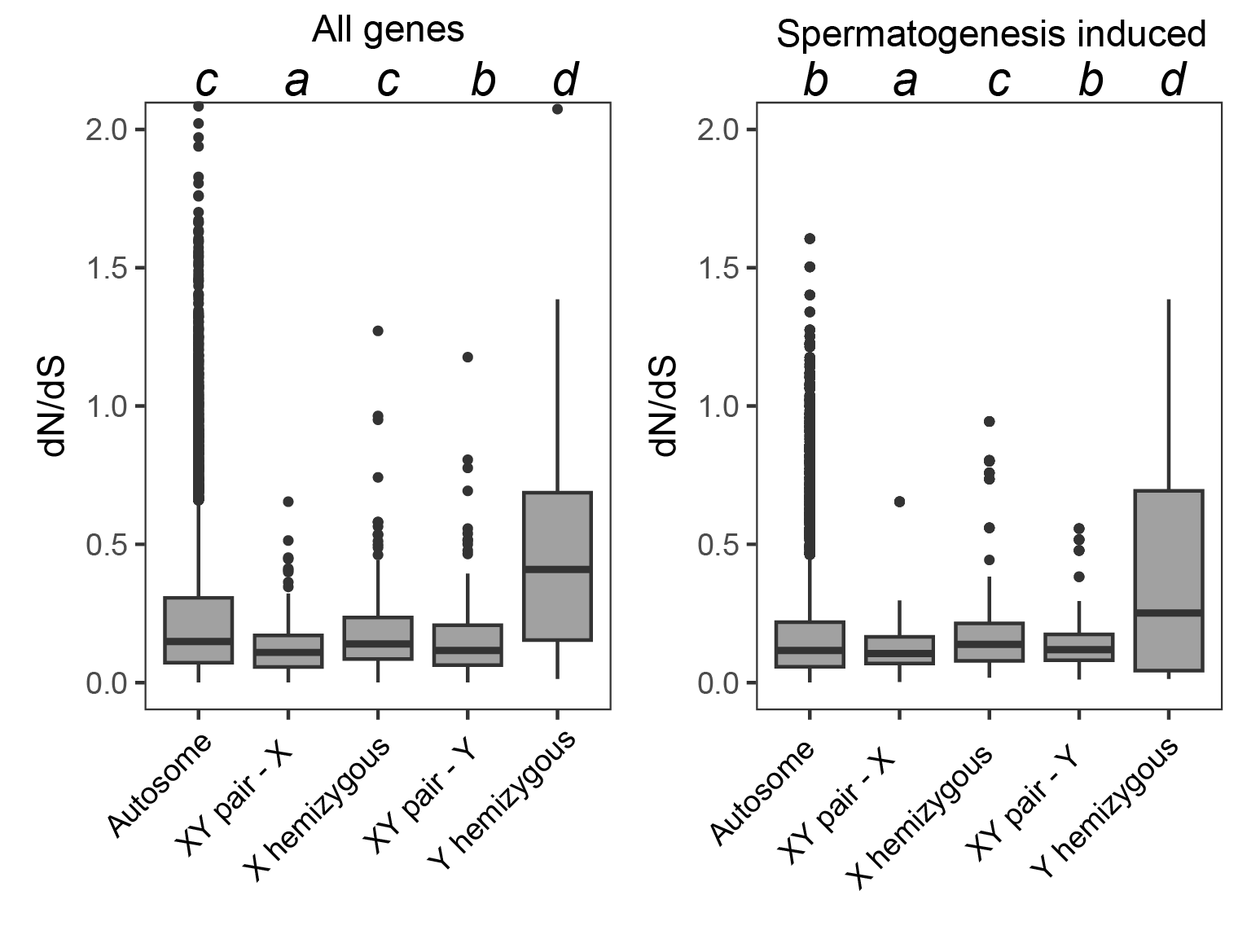

Supplement: S10 Fig — dN/dS was calculated between ninespine and threespine stickleback for genes on autosomes, X hemizygous genes, X and Y gametologs, and Y homozygous genes. scRNA-seq was used to identify genes that were induced in four major cell groups. Letters denote statistical comparisons across all remaining stages and categories based on Kruskal Wallis and Dunn’s test. Autosomes have the highest count with 2,262 genes followed by X hemizygous genes with 50 genes, X gametologs with 31 genes, Y gametologs with 31 genes, and Y hemizygous genes with just 4 genes. (TIF) [file pgen.1011875.s010.tif]

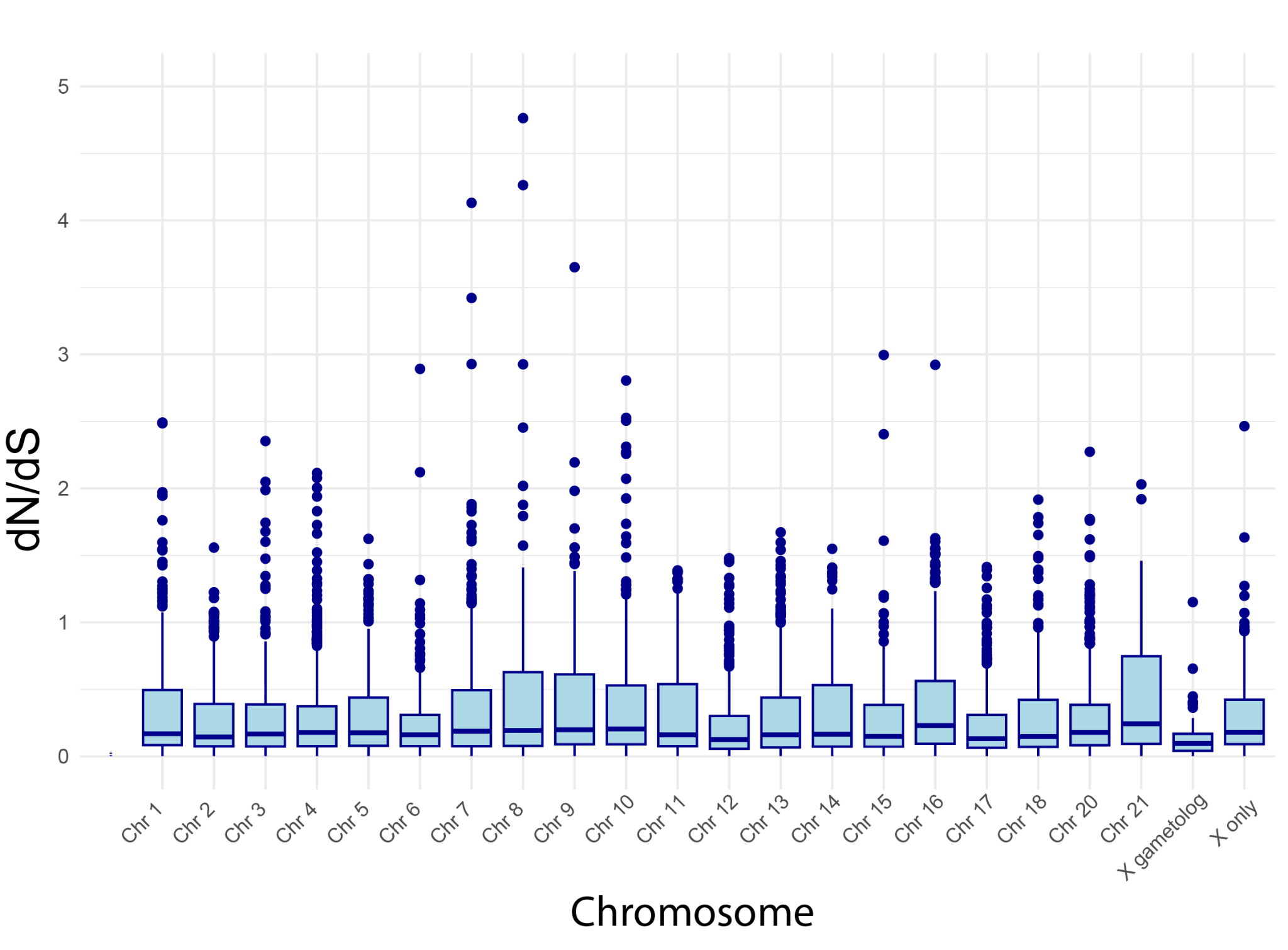

Supplement: S11 Fig — Both X gametologs and Chr 12 (Ninespine sex chromosomes) have lower dN/dS than median autosomal (P < 0.01, Kruskal Wallis and Dunn’s test). All other comparisons are not statistically different (P > 0.05 for all pairwise comparisons). (TIF) [file pgen.1011875.s011.tif]

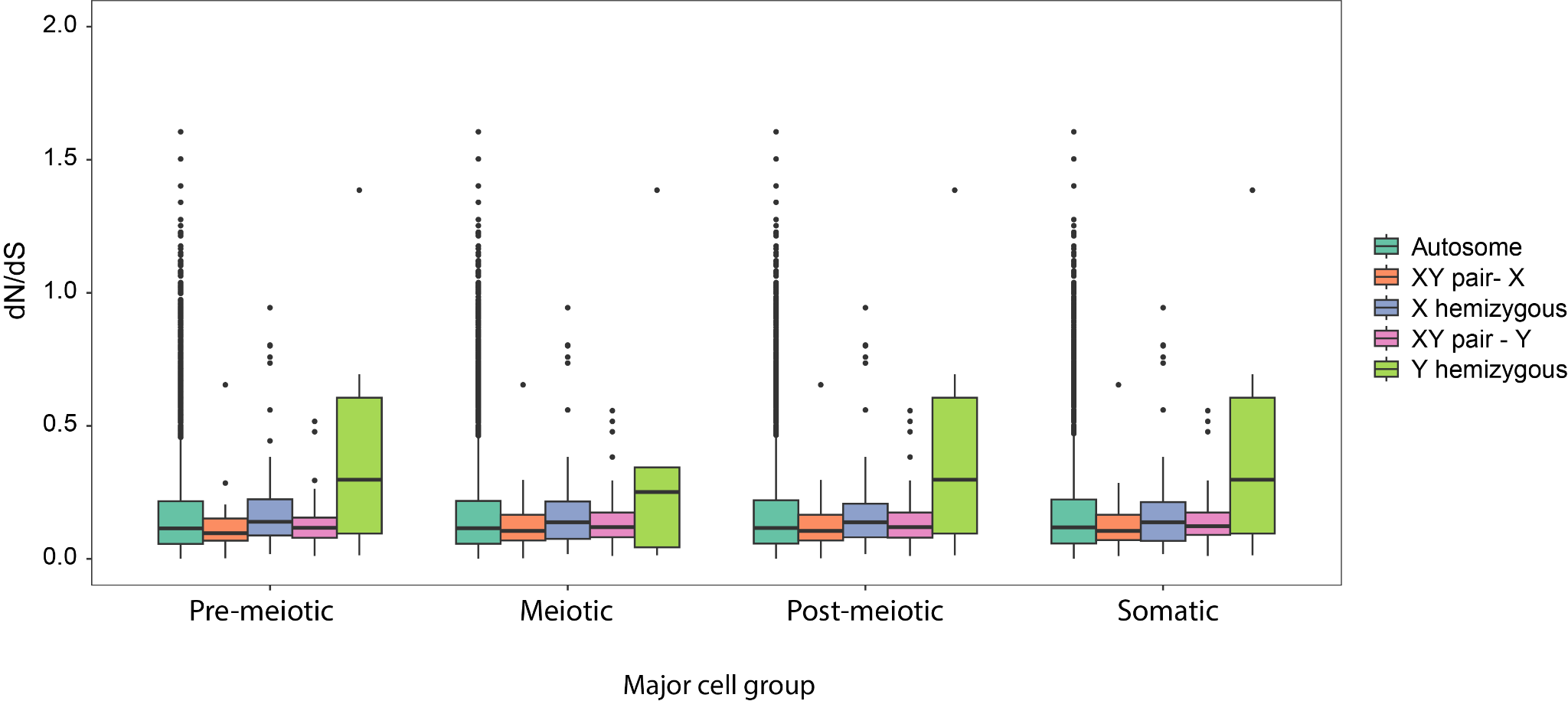

Supplement: S12 Fig — There were no significant differences between stages for each chromosome group (P = 0.501 Kruskal Wallis and Dunn’s test). The dataset includes genes that were induced and had an alignment between threespine and ninespine stickleback cross four developmental stages for each chromosome category. Autosomes have the largest sample sizes, with 414, 465, 340, and 1,043 genes across the four stages, respectively. X gametologs include 16, 3, 5, and 7 genes; X-only genes have 24, 9, 9, and 8 genes; Y gametologs consist of 16, 3, 5, and 7 genes; and Y-only genes are the smallest group, with just 2 genes in each of the last two stages. (TIF) [file pgen.1011875.s012.tif]

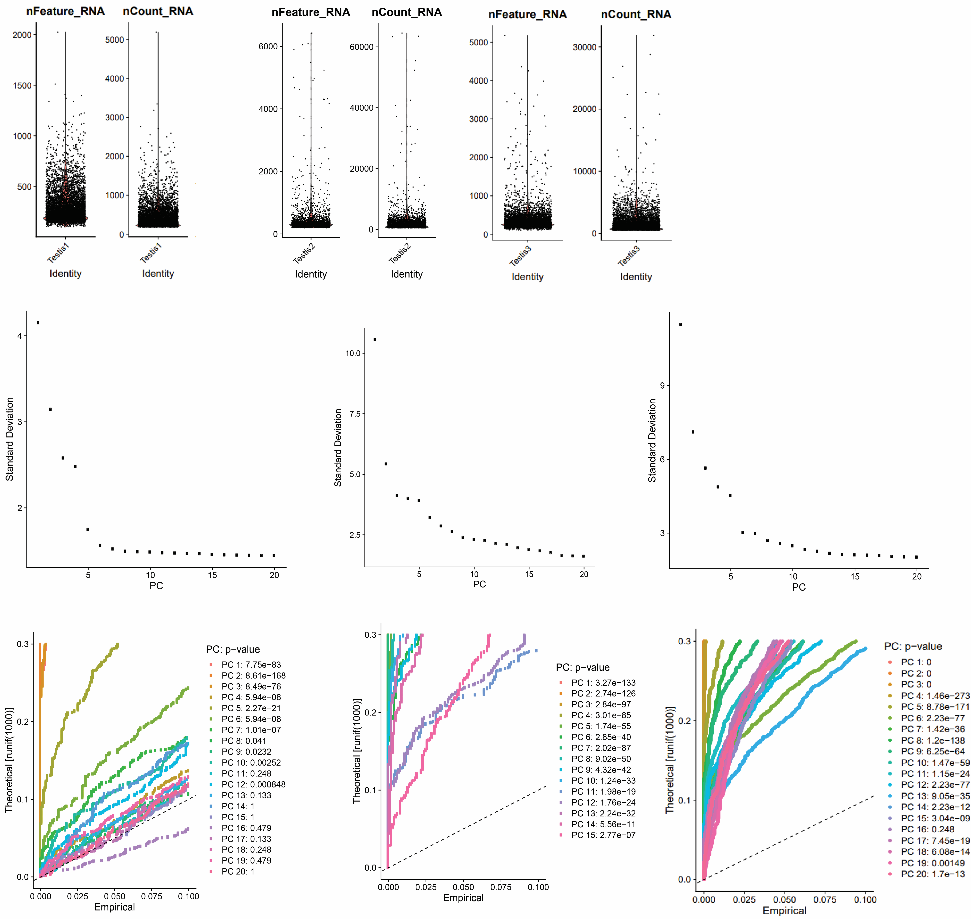

Supplement: S13 Fig — Elbow plots and jackstraw simulations were generated to assess the number of significant principle components for each sequencing experiment. (TIF) [file pgen.1011875.s013.tif]

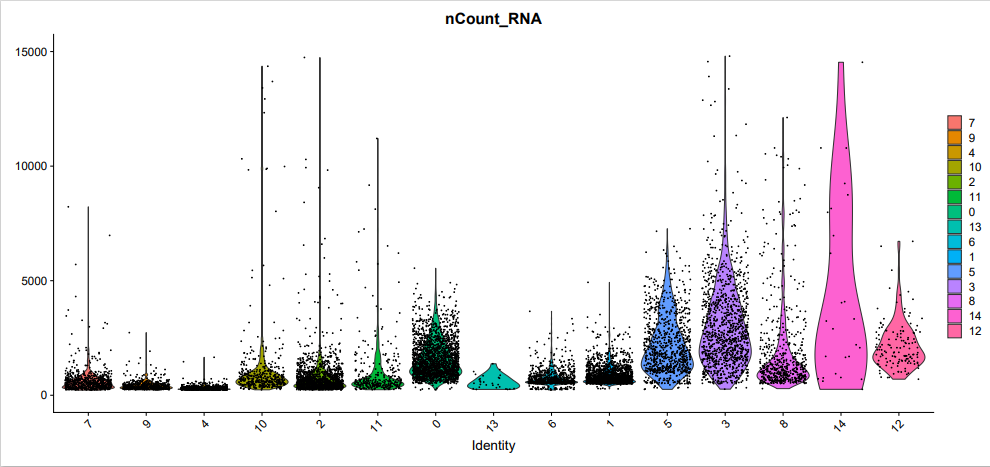

Supplement: S14 Fig — Number of RNA molecules identified per cell within each cell cluster were counted using Seurat. (TIF) [file pgen.1011875.s014.tif]
